# Operative versus non-operative treatment of ulnar styloid process base fractures: a systematic review and meta-analysis

**DOI:** 10.1007/s00068-024-02660-2

**Published:** 2024-09-13

**Authors:** L. X. van Rossenberg, F. J. P. Beeres, M. van Heijl, U. Hug, R. H. H. Groenwold, R. M. Houwert, B. J. M. van de Wall, The NEXT Study Group

**Affiliations:** 1 Department of Health Sciences and Medicine, University of Lucerne, Frohburgstrasse 3, 6002 Lucerne, Switzerland; 2 Department of Orthopaedic and Trauma Surgery, Lucerne Cantonal Hospital, Spitalstrasse 16, 6000 Lucerne, Switzerland; 3 The Diakonessenhuis Hospital Utrecht, Bosboomstraat 1, 3582 KE Utrecht, The Netherlands; 4 Leiden University Medical Center, Albinusdreef 2, 2333 ZA Leiden, The Netherlands; 5 University Medical Center Utrecht, Heidelberglaan 8, 3584 CS Utrecht, The Netherlands

**Keywords:** Ulnar styloid process (USP), Distal radius fracture (DRF), Tension band wiring (TBW), Distal radio-ulnar joint (DRUJ), Ulnar styloid base fracture

## Abstract

**Purpose:**

Ulnar styloid process (USP) fractures are present in 40–65% of all distal radius fractures (DRFs). USP base fractures can be associated with distal radioulnar joint (DRUJ) instability and ulnar sided wrist pain and are treated by conservative management and surgical fixation, without consensus. This systematic review and meta-analysis compares operative to non-operative treatment of concomitant ulnar styloid base fractures in patients with distal radius fractures.

**Methods:**

PubMed/Medline/Embase/CENTRAL databases were searched identifying RCTs and comparative observational studies. Effect estimates were extracted and pooled using random effect models to account for heterogeneity across studies. Results were presented as (standardized) mean differences (SMD or MD) or odds ratios (OR) and corresponding 95% confidence intervals (95%CI).

**Results:**

Two RCTs (161 patients) and three observational studies (175 patients) were included. Tension band wiring was used for surgically treated USP fractures. Results were comparable across the different study designs and hence pooled across studies. Non-surgically treated patients had better wrist function at 6 months (SMD 0.57, 95%CI 0.30; 0.90, I^2^ = 0%). After 12 months there was no observed difference (MD 2.31, 95%CI −2.57; 7.19, I^2^ = 91%). Fewer patients had USP non-unions in the operative group (OR 0.08, 95%CI 0.04; 0.18, I^2^ = 0%). More patients suffered complications in the operative group (OR 14.3; 95%CI 1.08; 188, I^2^ = 89%).

**Conclusion:**

Routinely fixating USP base fractures as standard of care is not indicated. Surgery may be considered in selective cases (e.g. persistent DRUJ instability during ballottement test after fixation of the radius).

**Supplementary Information:**

The online version contains supplementary material available at 10.1007/s00068-024-02660-2.

## Introduction

Ulnar styloid process (USP) fractures are present in 40–65% of all distal radius fractures (DRFs) [1–7]. Typically USP fractures are classified by location as either a tip, waist or base fractures and are often ignored when the DRF can be managed conservatively [8–10]. While tip and waist fractures bear almost no clinical relevance, base fractures can be associated with distal radioulnar joint (DRUJ) instability and ulnar sided wrist pain due to triangular fibrocartilage complex (TFCC) tears and non-union [8, 10–14].

To date, it remains unclear whether USP base fractures should be surgically stabilised in patients with a distal radius fracture and concomitant USP base fracture. Several studies suggest that USP base fractures do not negatively influence functional results or the development of chronic DRUJ instability [7, 15, 16]. Additionally, studies report that in most cases near anatomical reduction followed by volar plate fixation of the distal radius prevents DRUJ instability regardless of the presence of a USP fracture [9, 17–20]. On the other hand, some studies report higher rates of ulnar sided wrist pain due to non-union of the USP indicating a need for ${\dot{\text{V}}}$ fixation [8, 14, 21].

Recently, multiple comparative studies regarding operative versus non-operative management of the ulnar styloid in patients with distal radius fractures have been published, allowing meta-analysis of the data [22–26]. The aim of this systematic review and meta-analysis was to compare operative to non-operative management of concomitant ulnar styloid process base fractures in adult patients with surgically managed distal radius fractures. The primary outcome was patient reported wrist function. Secondary outcomes included bony union of the ulnar styloid, grip strength, range of motion, pain scores, and complications.

## Methods

This systematic review and meta-analysis was reported in accordance with the Preferred Reporting Items for Systematic Reviews and Meta-analysis (PRISMA) guidelines and Meta-analysis of Observational Studies in Epidemiology checklist (MOOSE) [27, 28]. The study protocol was registered on the PROSPERO platform prior to completion of the study (registration number CRD42023432739). Ethical approval was not required for this systematic review and meta-analysis.

### Search strategy and selection criteria

The PubMed/Medline, Embase and CENTRAL databases were searched on 31 March 2023, for comparative studies on operative versus non-operative treatment of concomitant USP fractures in patients with DRF’s. The search was updated on 7 November 2023. The search syntax is presented in supplementary materials Table 1. Two reviewers (LXvR, BJMvdW) independently performed title, abstract, and full text screening for study eligibility using Rayyan software. Inclusion criteria were comparison of operative versus non-operative treatment of concomitant USP fractures in patients with distal radius fractures, age ≥ 18 years and reporting outcomes of interest (wrist function, bony union, grip strength, range of motion, pain scores and complications). Both randomized controlled trials and comparative observational studies were considered eligible for inclusion. Exclusion criteria were other study designs than RCT’s or comparative observational studies, no full text availability, no USP base fractures included, mean follow up less than 3 months, and language other than Dutch, English, or German. Disagreement was resolved through interrater discussion. In case of unresolved disagreement, a third reviewer (FJPB) was consulted. All included studies were cross referenced to identify any additional studies not accounted for in the original search.

**Table 1:** Baseline characteristics of studies comparing operative to conservative management of ulnar styloid process fractures

Author	Year	Country	Design	Study period	Mean follow-up (SD/range)	Type of fixation	Total number of patients		Mean age (SD or range)		Gender, male (%)		USP base fracture (%)		DRF AO classification (A/B/C)	
							OP	NONOP	OP	NONOP	OP	NONOP	OP	NONOP	OP	NONOP
Zenke	2012	Japan	Pros	2006–2008	12.5 (7.3) months	TBW	20	28	63.7 (15.5)	64.3 (18.2)	2 (10.0%)	4 (14.3%)	20 (100%)	28 (100%)	10/1/9	13/2/13
Sawada	2016	Japan	Pros	2006–2007 and 2010–2011	26.8 (10–55) weeks	Pinning/TBW/Acutwist	16	48	58.9 (33–78)	61.1 (38–74)	7 (43.8%)	15 (31.3%)	11 (68.8%)	31 (64.4%)	5/2/9	18/1/29
Moradi	2021	Iran	RCT	2016–2017	*N.r.* (3–12 months)	TBW	39	36	51.5 (14.8)	52.9 (16.2)	26 (66.7%)	17 (47.2%)	39 (100%)	36 (100%)	39/0/0	36/0/0
Afifi	2022	Egypt	RCT	2015–2021	24 (16–38) months	TBW	43	43	35 (5.1)	34 (5.1)	36 (83.7%)	30 (69.8%)	43 (100%)	43 (100%)	12/10/21	16/7/20
Velmurugesan	2023	India	Retro	2018–2019	*N.r.* (range 24–36 months)	TBW	14	49 (matched analysis* n* = 28)	40.3 (12.7)	43.3 (14.8)	51^a^ (81.0%)		14 (100%)	49 (100%)	9/41/13^a^	

*N.r.* not reported, *Pros* prospective, *Retro* retrospective, *RCT* randomized controlled trial, *TBW* tension band wiring

^a^Value reported only for the total group

### Data extraction

Data extraction was performed independently by two reviewers (LXvR, BJMvdW) using a predefined data extraction file for baseline characteristics including first author, year of publication, study period, country, study design and number of participants. Patient characteristics of participants were collected including age, sex, duration of follow up, type of USP fracture, type of DRF, and surgical fixation technique. USP fractures were classified as base, waist or tip fractures. DRF’s were classified by the AO classification. In case other classifications (e.g. Fernandez) were used, scores were converted to the AO format.

### Quality assessment

Quality assessment was performed independently by two reviewers (LXvR, BJMvdW) using the recently developed NEXT-critical appraisal tool for RCT’s and observational studies [29]. Studies are scored on nine items of which four relate to the PICO applicability and five to the quality of the methodology. Each item was independently assessed and scored as either 0 (poor applicability), 1 (moderate applicability) or 2 (good applicability). Disagreement was resolved through interrater discussion or consultation of a third reviewer (FJPB). A detailed overview of the methodological quality assessment per item and an overall score is provided in supplement Table 2.

**Table 2:** Complications reported in included studies comparing operative and non-operative management of ulnar styloid process fractures

Study (total complications)	Afifi et al. (*n* = 42)		Zenke et al. (*n* = 8)		Velmurugesan et al. (*n* = 19)	
Treatment group (patients per group)	Operative (*n* = 43)	Non-operative (*n* = 43)	Operative (*n* = 20)	Non-operative (*n* = 28)	Operative (*n* = 14)	Non-operative (*n* = 49)
Type of complications						
Hardware irritation	31 (73.8%)	*n.r.*	5 (62.5%)	1 (12.5%)^a^	2 (10.5%)	*n.r.*
Hardware failure (loosening screw of volar plate)	*n.r.*	*n.r.*	*n.r.*	1 (12.5%)	*n.r.*	*n.r.*
Temporary paresthesia of the DBUN	6 (14.3%)	*n.r.*	*n.r.*	*n.r.*	1 (5.3%)	*n.r.*
Persistant ulnar wrist pain	3 (7.1%)	*n.r.*	*n.r.*	*n.r.*	5 (26.3%)	11 (57.9%)
DRUJ instability	*n.r.*	2 (4.8%)	*n.r.*	*n.r.*	*n.r.*	*n.r.*
Partial injury of superficial palmar branch of median nerve	*n.r.*	*n.r.*	*n.r.*	1 (12.5%)	0	*n.r.*
^b^Complications for which secondary surgery was required	26 (61.9%)	1 (2.4%)	5 (62.5%)	1 (12.5%)	2 (10.5%)	0
^c^Total complications per treatment group (%)	40 (93.0%)	2 (4.6%)	5 (25.0%)	3 (10.7%)	8 (57.1%)	11 (22.4%)

*N.r.* not reported, *DBUN* dorsal branch of ulnar nerve

^a^Hardware irritation of the radial plate

^b^Types of surgery by frequency: Removal of hardware (*n* = 30); Ulnar styloidectomy (*n* = 3); Styloidectomy and TFCC reinsertion (*n* = 1); Neurolysis of superficial palmar branch of median nerve (*n* = 1)

^c^Number (%) of patients in the treatment group with complications

### Study outcomes

The primary outcome was patient reported wrist function. All upper extremity functional scores (Disability of Shoulder and Hand (DASH), quick-DASH, Hand20 and Modified Mayo questionnaires) were standardized and pooled for 6 months and more than 12 months after injury.

Secondary outcomes included radiographic union of the USP fracture, grip strength, range of motion, pain scores, and complications. Non-union was defined as the absence of bridging trabeculae at >6 months. When possible, distinction was made whether non-unions were symptomatic (pain complaints in fracture zone during movement) or not. Grip strength was measured with a dynamometer, range of motion with a goniometer and pain scores with the visual analogue scale at >6 months after injury.

### Statistical analysis

Meta-analysis was conducted using Review Manager (RevMan, version 5.4.1). Information regarding continuous data was presented as means with standard deviations (SD) or range. In case extracted information was described differently between studies, information was converted to mean (SD) using the methods described in the Cochrane Handbook for Systematic Reviews of Interventions [30]. Information regarding dichotomous variables was presented as counts and percentages. Treatment effects for continuous variables were pooled using the random effects inverse variance weighting method and binary outcomes using the random effects Mantel–Haenszel method. All outcomes were presented as either weighted odds ratio (OR), weighted mean difference (MD) or standardized weighted mean difference (SMD) with corresponding 95% confidence intervals (95%CI). Heterogeneity among studies was assessed by inspection of forest plots and quantified by the I^2^ statistic. Stratification according to study design was not performed due to the low number of included studies. Significance level was set at a *p*-value threshold of 0.05.

## Results

### Search results

**Fig. 1:**
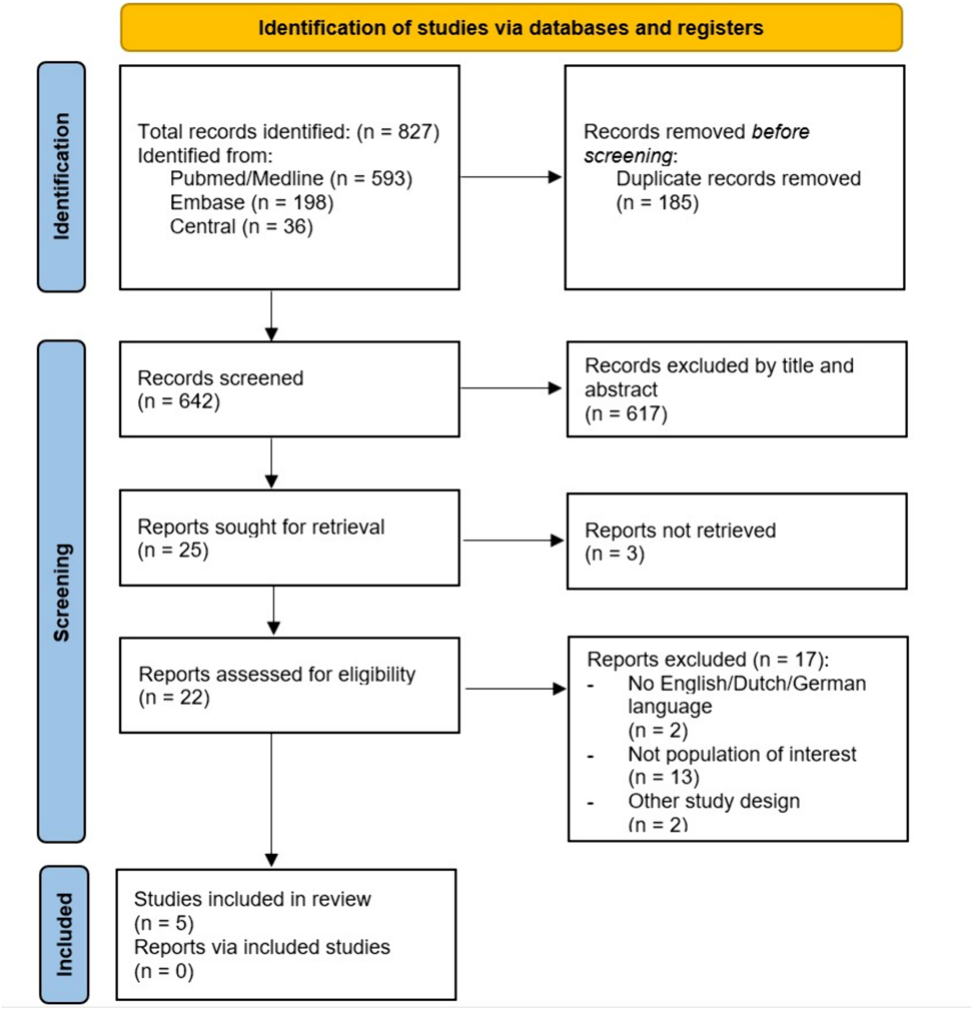
PRISMA flow chart representing the search and screening process of articles comparing operative to non-operative treatment of ulnar styloid process fractures. Format by Page et al. [31]

The flowchart of the literature search and study selection is depicted in Fig. 1. A total of five studies were included for analysis of which two were randomized controlled trials and three observational studies [22–26].

### Study characteristics

In the five studies, a total of 336 patients were included. Of all patients, 132 were treated operatively for the USP fracture and 204 non-operatively. Four studies included only USP base fractures [22, 23, 25, 26], while one study included USP base, waist and tip fractures [24]. The reason for including the latter was because the majority of these fractures in the operative (68.8%) and non-operative group (64.6%) were base fractures and the pooled estimate of the primary outcome did not change when this study was removed from the analysis (SMD 0.57; 95%CI 0.26–0.88; I^2^ = 0% versus SMD 0.65; 95%CI 0.28–1.02; I^2^ = 0%) [24]. None of the included observational studies corrected for confounding, however, pooled estimates for observational studies and RCT’s were similar. Distributions of baseline characteristics were similar across treatment groups in each study. All baseline characteristics are provided in Table 1.

### Quality assessment

The mean critical appraisal score of included studies was 12.2 (range 10–16); 14.5 (range 13–16) for RCT’s was and 10.6 (range 10–11) for observational studies (supplement Table 2). Hence, overall applicability of the included studies was moderate to good. Items on which all performed well include item 1 “Is the patient population included in the study representative of the patient population defined in the PICO of the systematic review?” and item 7 “Was the intervention status correctly classified?”. Items on which studies performed poorly include item 5 “Is there comparability of intervention groups, or are appropriate methods applied to correct for incomparability?” and item 9 “Were analyses prespecified and did the study adhere to the specified analysis plan?”. Since none of the observational studies adequately corrected for confounding, the estimated relations between treatment and outcome are unadjusted for potential confounding. However, pooled estimates of RCT’s and observational studies are similar. An overview of scores per item is presented in supplement Fig. 1.

### Primary outcome

**Fig. 2:**
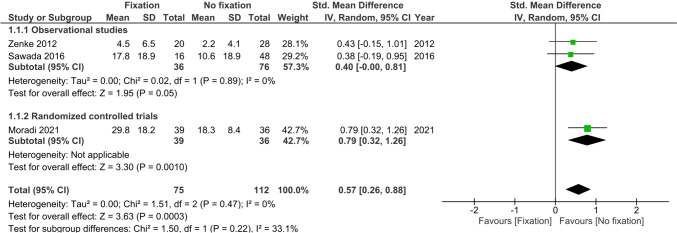
Forest plot of wrist function at 6 months after operative versus non-operative management of ulnar styloid process fractures

Functional wrist scores at 6 months were reported in three studies (*n* = 187) [23, 24, 26]. A difference in wrist function at 6 months was observed in favour of the non-operative group (SMD 0.57, 95%CI 0.26; 0.88, I^2^ = 0%) (Fig. 2).

**Fig. 3:**
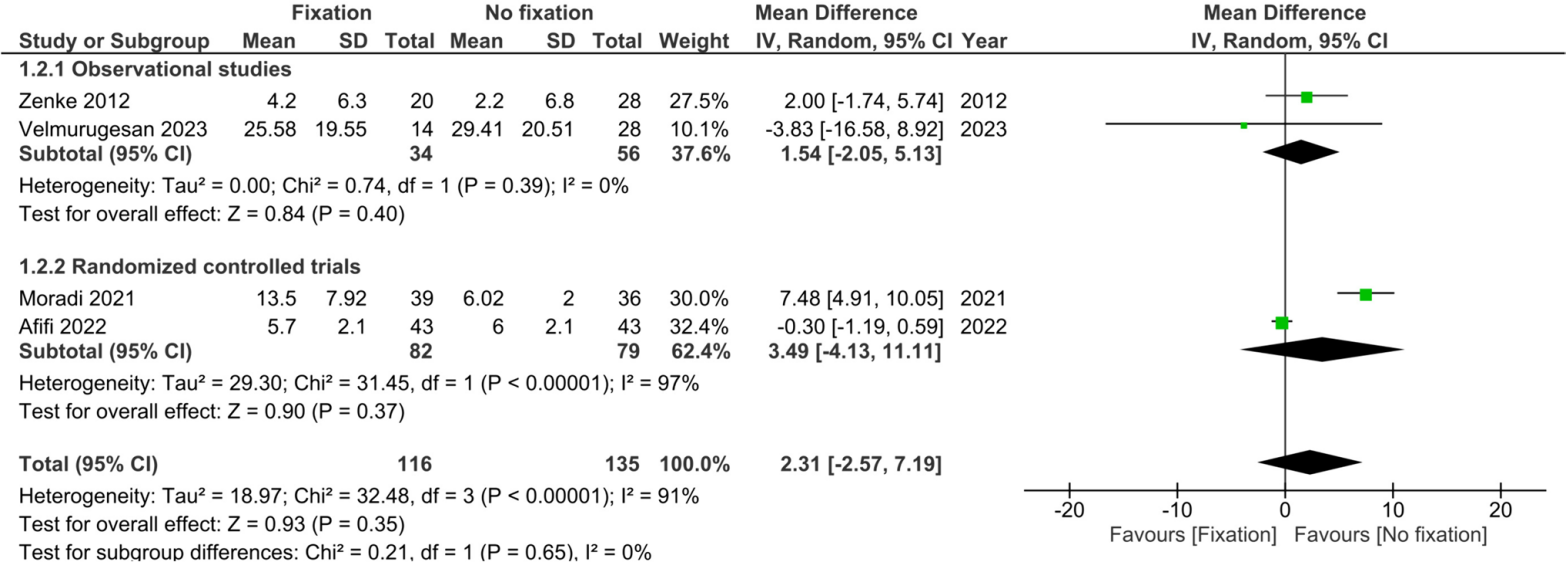
Forest plot of wrist function at 12 or more months after operative versus non-operative management of ulnar styloid process fractures

Four studies (*n* = 251) reported functional wrist scores using the (quick)-DASH questionnaire at >12 months [22, 23, 25, 26]. No difference was observed in wrist function at >12 months between treatment groups (MD 2.31, 95%CI −2.57; 7.19, I^2^ = 91%) (Fig. 3).

### Secondary outcomes

**Fig. 4:**
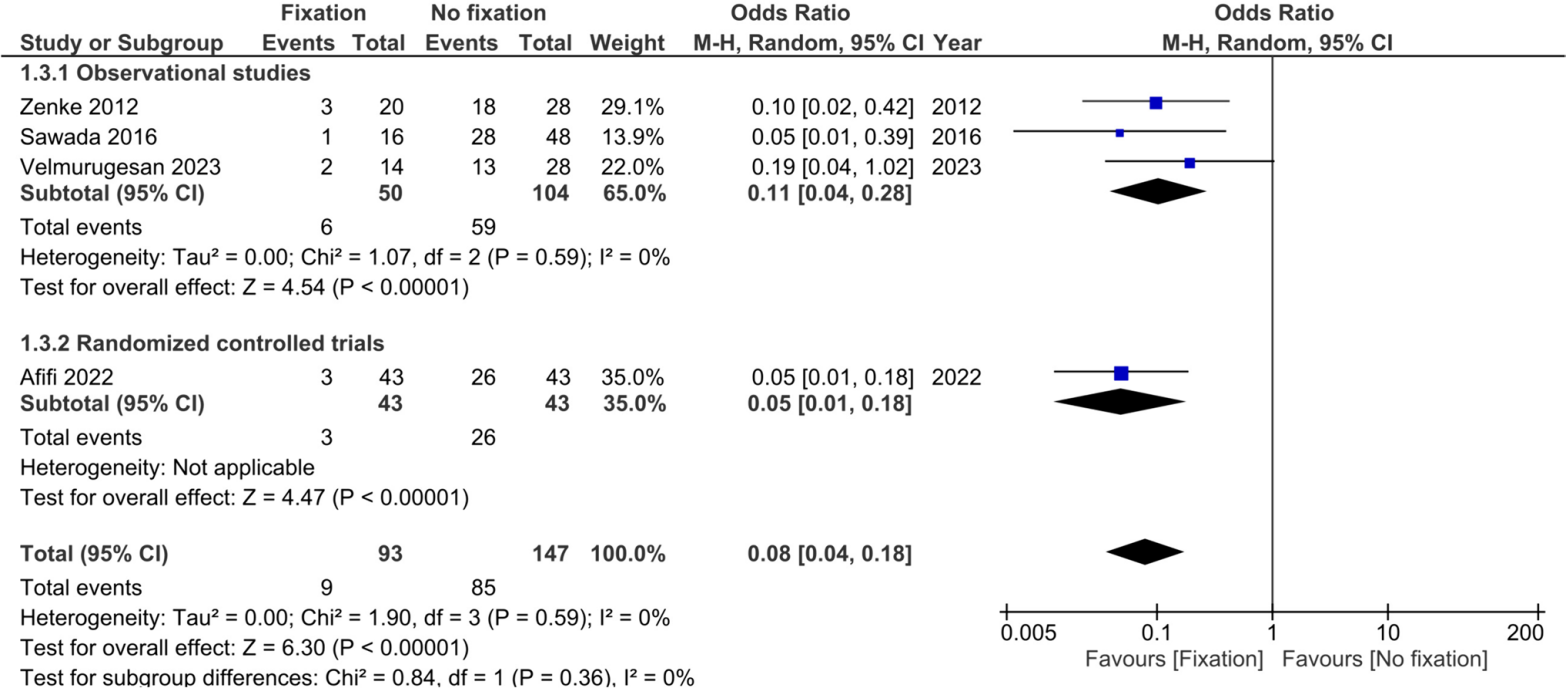
Forest plot of non-union events after operative versus non-operative management of ulnar styloid process fractures

Non-union of the USP was reported in four studies (*n* = 240) and was present in 9.7% of fractures in the operative group versus 57.8% in the non-operative group [22, 24–26]. The pooled estimate showed a difference in favour of the operative group (OR 0.08, 95%CI 0.04; 0.18, I^2^ = 0%) (Fig. 4).

Only two out of the four studies (with a combined sample size of 71 patients in the non-operative and 57 patients in the operative group) reported whether non-unions were symptomatic [22, 25]. A total of 44 non-unions were reported in these studies, 39 (54.9%) in the non-operative group and five (8.8%) in the operative group. Nine (12.7%) non-unions were symptomatic in the non-operative group versus five (8.8%) in the operative group. In the non-operative group seven of the patients with symptomatic non-unions had ulnar sided wrist pain and two had persistent DRUJ instability. One patient with DRUJ instability required ulnar styloidectomy and TFCC foveal reinsertion after which symptoms resolved. In the operative group all five of the patients with symptomatic non-unions had ulnar sided wrist pain without DRUJ instability. No distinction could be made whether symptoms occurred due to non-union or hardware irritation. Three patients required hardware removal and ulnar styloidectomy after which symptoms resolved [22, 25]. The other two studies reported no correlation between non-union and ulnar sided wrist pain [24, 26].

**Fig. 5:**
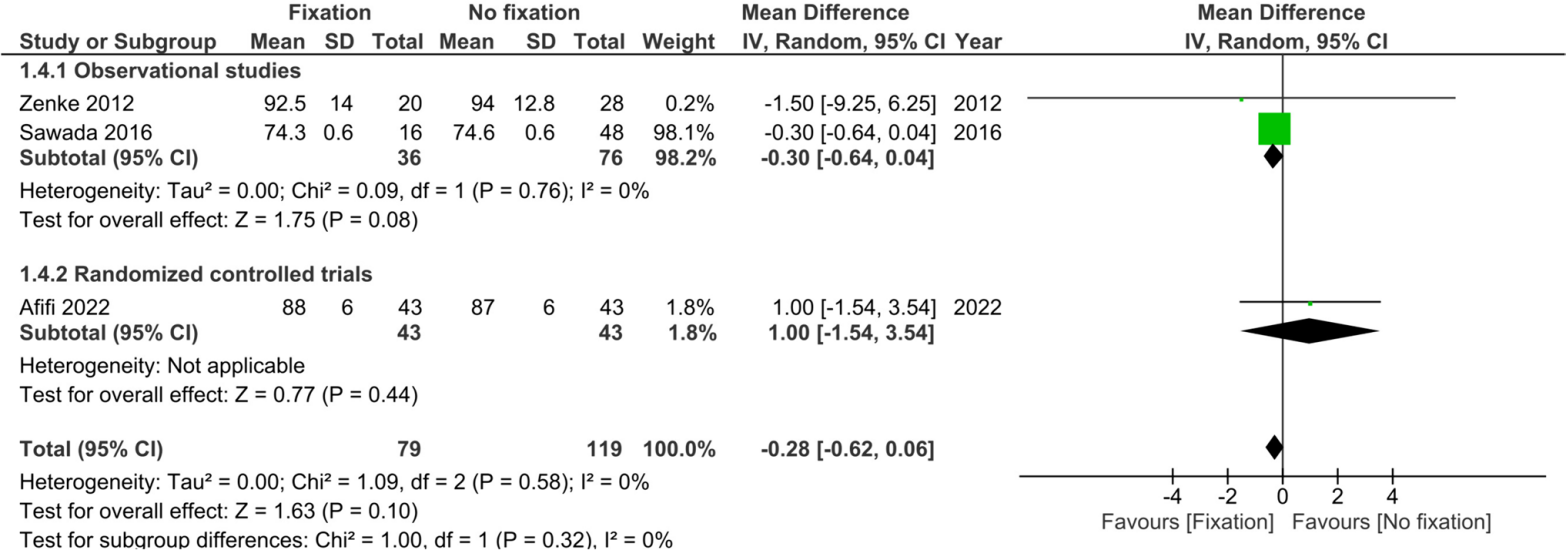
Forest plot of grip strength at six or more months after operative versus non-operative management of ulnar styloid process fractures

Three studies (*n* = 198) reported grip strength at >6 months after injury [22, 24, 26]. No difference was observed in grip strength among treatment groups (MD −0.28%, 95%CI −0.62; 0.06, I^2^ = 0%), see Fig. 5.

**Fig. 6:**
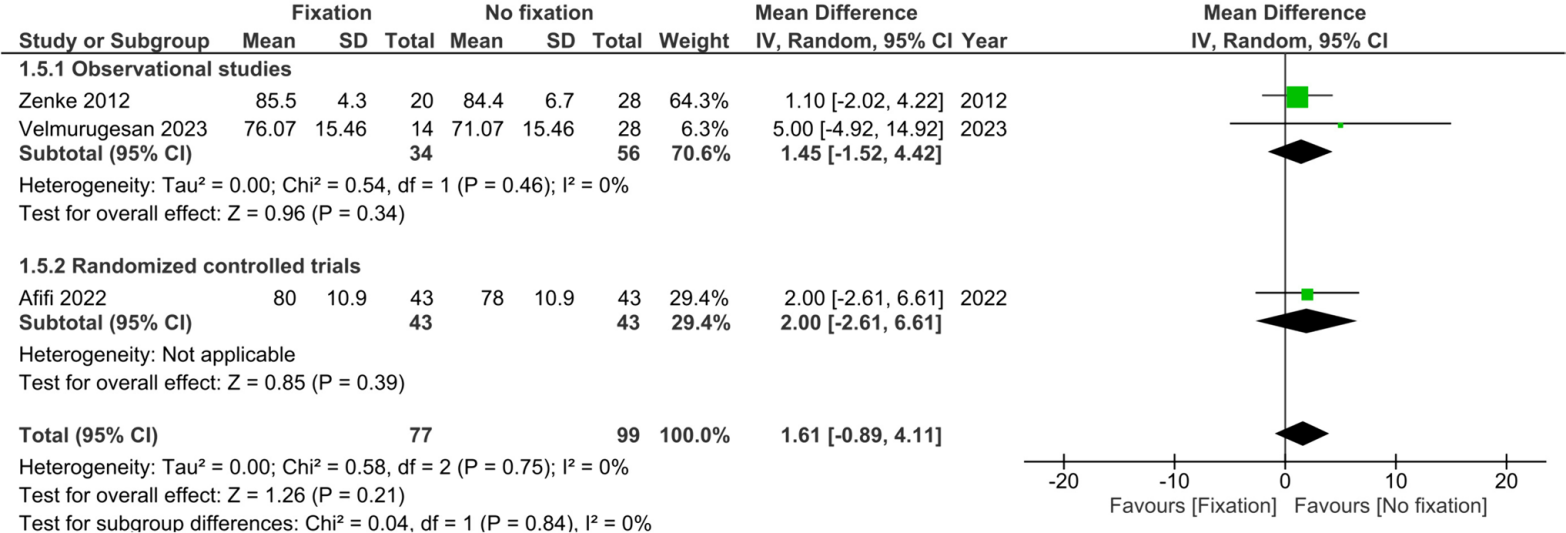
Forest plot of flexion at six or more months after operative versus non-operative management of ulnar styloid process fractures

**Fig. 7:**
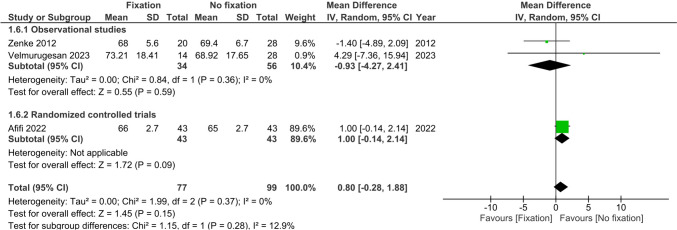
Forest plot of extension at six or more months after operative versus non-operative management of ulnar styloid process fractures

**Fig. 8:**
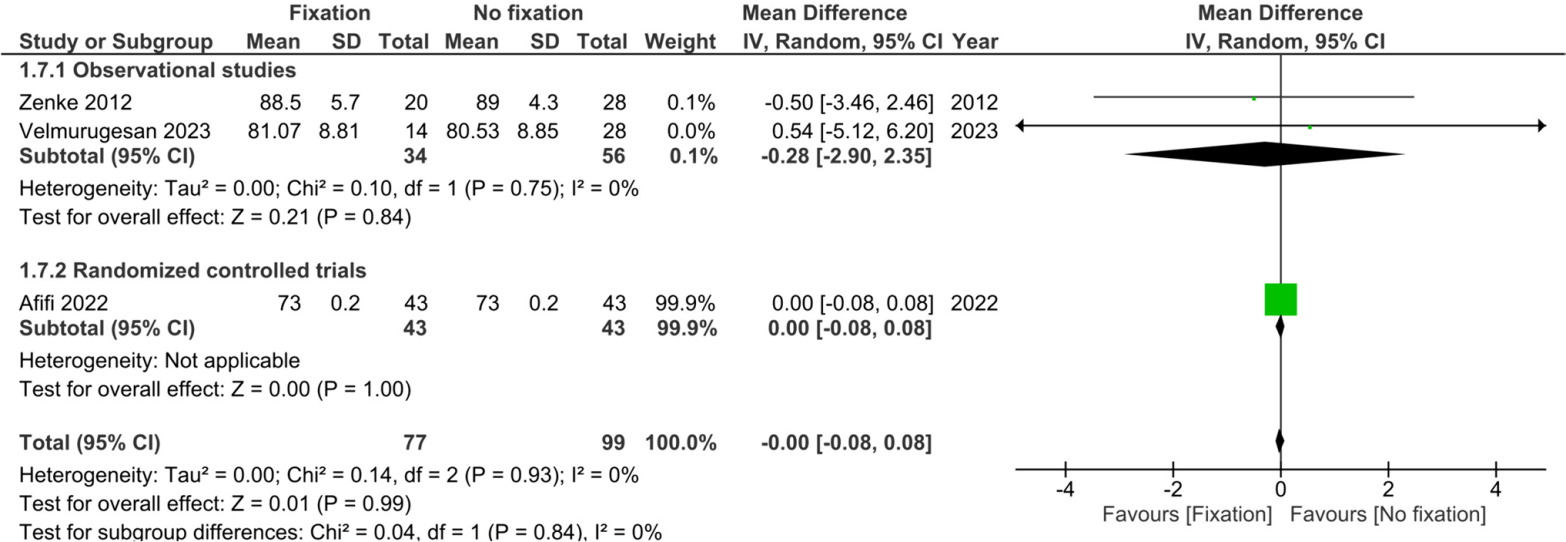
Forest plot of supination at six or more months after operative versus non-operative management of ulnar styloid process fractures

**Fig. 9:**
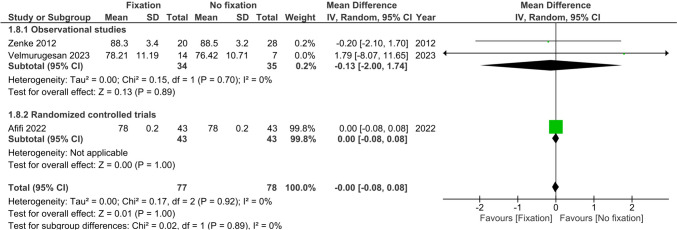
Forest plot of pronation at six or more months after operative versus non-operative management of ulnar styloid process fractures

Range of motion (flexion, extension, supination and pronation) at >6 months was reported by three studies (*n* = 176) [22, 25, 26]. There were no differences observed between treatment groups in flexion (MD 1.61°, 95%CI −0.89; 4.11, I^2^ = 0%), extension (MD 0.80°, 95%CI −0.28; 1.88, I^2^ = 0%), supination (MD 0.0°, 95%CI −0.08; 0.08, I^2^ = 0%) and pronation (MD 0.0°, 95%CI −0.08; 0.08, I^2^ = 0%), see Figs. 6, 7, 8 and 9.

**Fig. 10:**
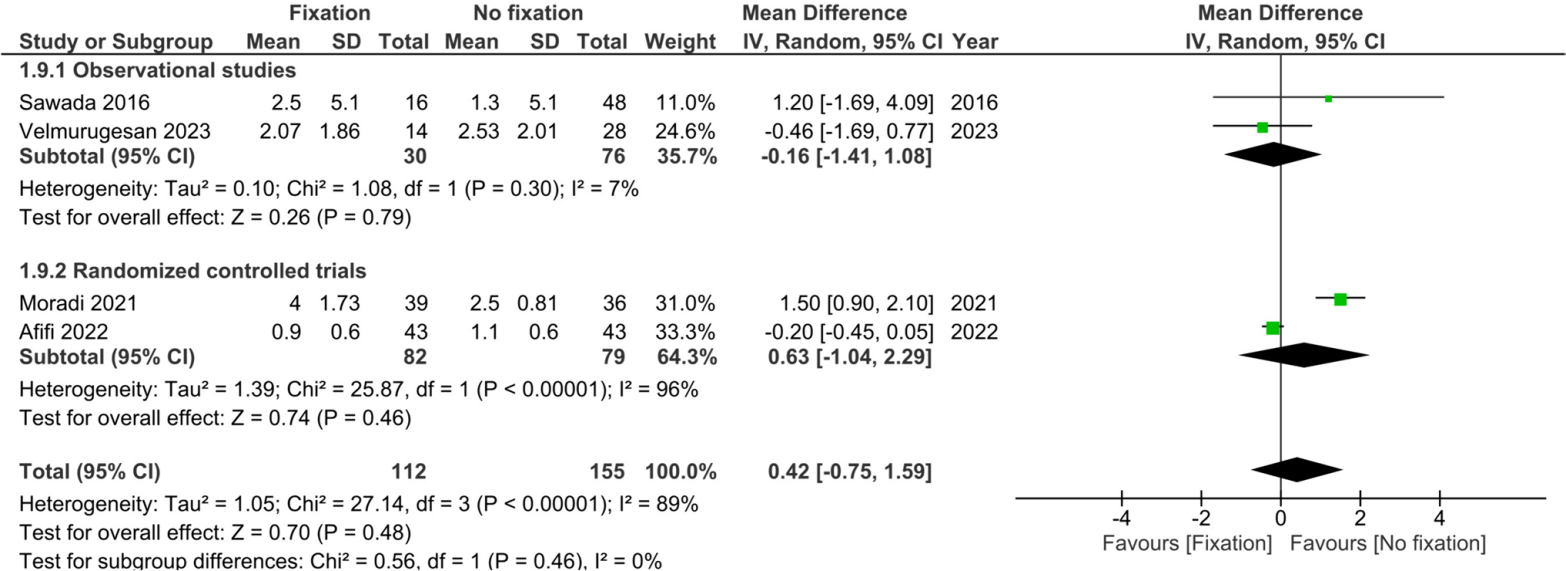
Forest plot of pain scores at six or more months after operative versus non-operative management of ulnar styloid process fractures

Four studies (*n* = 225) reported pain scores (VAS) at >6 months after injury [22–25]. No difference was observed in pain scores after >6 months between treatment groups (MD 0.42, 95%CI −0.75; 1.59, I^2^ = 92%), see Fig. 10.

**Fig. 11:**
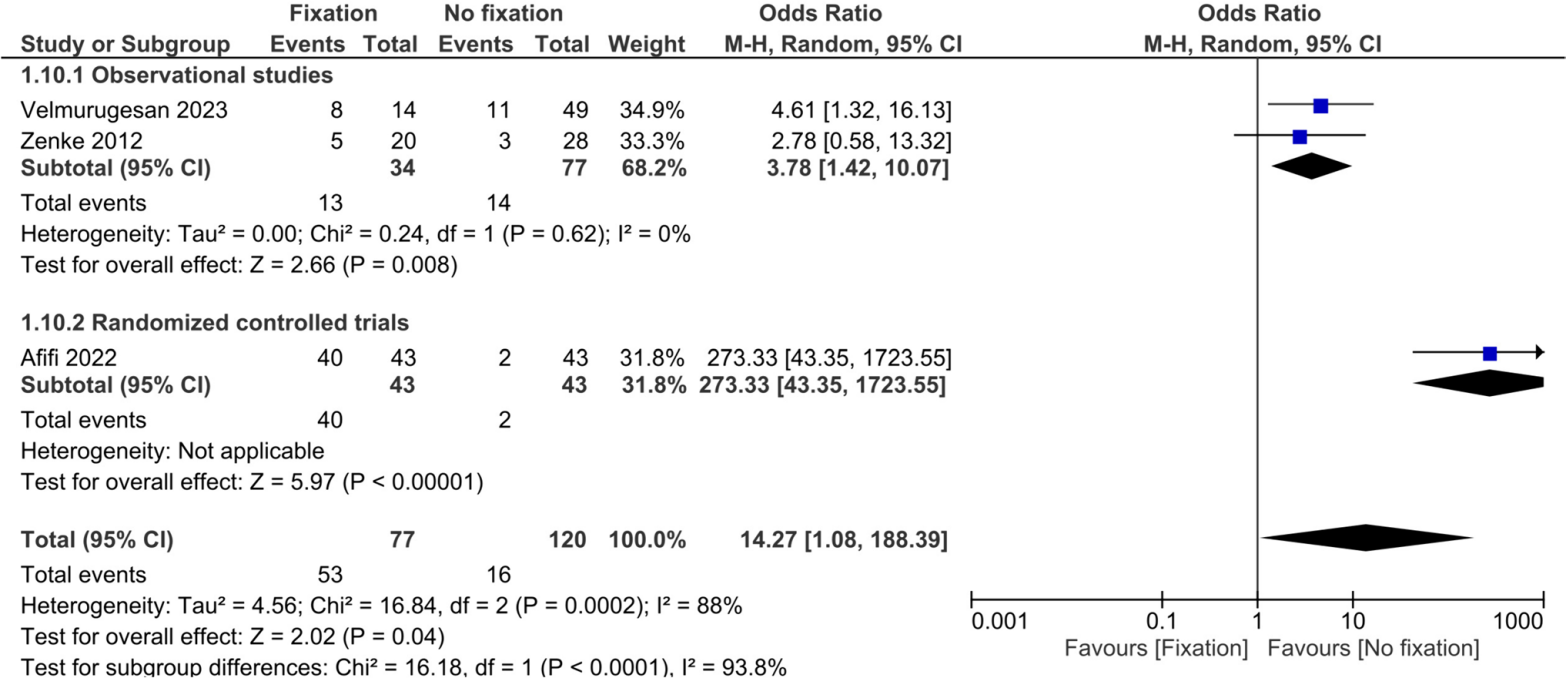
Forest plot of complication events after operative versus non-operative management of ulnar styloid process fractures

Three studies reported complications and were present in 53 (68.8%) of cases in the operative group versus 16 (13.3%) in the non-operative group, see Table 2 [22, 25, 26]. Complications occurred 3.3 times more frequently in the operative group (OR 14.3, 95%CI 1.08; 188, I^2^ = 88%), see Fig. 11.

In the operative group, hardware irritation occurred most frequently (*n* = 39, 57%), while in the non-operative group, ulnar sided wrist pain occurred most frequently (*n* = 19, 28%). See Table 2 for a detailed overview of complications and Table 3 for the Clavien–Dindo type distribution.

**Table 3:** Clavien–Dindo score of reported complications

Degree	Definition	Operative	Non-operative	Total (%)^c^
I	^a^Any deviation from the normal postoperative course withoud need of intervention beyond the administration ^b^medication and psychical therapy	20	14	34 (10.1%)
II	Complications requiring pharmacological treatment with other medicines beyond the ones used for complications of degree I	–	–	0
III	Complications requiring surgical, endoscopic or radiologic intervention	33	2	35 (10.4%)
IV	Life threatening complication requiring IC admission	–	–	0
V	Death	–	-	0

^a^This degree also includes wound infections opened at the bedside

^b^Medication include anti-emetics, antipyretics, analgesics, diuretics and electrolytes

^c^Percentage of complications in the total amount of patients (*n* = 336)

## Discussion

This systematic review and meta-analysis of two randomized controlled trials and three comparative observational studies describes the outcomes of operative and non-operative treatment of concomitant USP base fractures in adult patients with operatively managed distal radius fractures. Patient reported wrist function at 6 months after surgery was better in the non-operative group, while wrist function at >12 months was comparable. Non-union occurred more frequently in the non-operative group, however, most non-union were asymptomatic. Whether non-unions were symptomatic or not was reported in two of the included studies, which found most non-unions to be asymptomatic. Complications occurred more frequently in the operative group, predominantly caused by implant irritation and subsequent removal. There were no differences in grip strength, pain scores and range of motion at >12 months.

### Comparison to previous literature

To our knowledge, there currently is no systematic review comparing operative versus non-operative treatment of concomitant USP base fractures in patients with operatively managed distal radius fractures. However, two meta-analyses have been published comparing outcomes of distal radius fractures with and without concomitant USP fractures [2, 32]. The most recent meta-analysis by Mulders et al. was published in 2018 and analysed a total of 2243 patients [2]. They reported better patient reported wrist function after 12 months for the group without USP fractures. Similar to the present study, both meta-analyses reported no difference regarding range of motion and grip strength. A third meta-analysis by Wijffels et al. compared outcomes of union versus non-union of the USP in patients with DRFs [3]. Comparable to our results, no differences were reported regarding patient reported wrist function at 12 months, range of motion and grip strength. Since the abovementioned meta-analyses encompass different treatment groups, comparability is limited. However, we may draw some conclusions from these meta-analyses. Firstly, USP fractures indeed have a negative impact on functional outcome of the wrist. Secondly, non-unions are often asymptomatic. Therefore, there might be a role for restoring anatomy using fixation techniques for USP base fractures. Although rather than routinely, this should be performed selectively.

### Interpretation of results

The present meta-analysis supports this line of thought. None of the findings indicate that routinely fixating USP base fractures is beneficial. Indeed, non-unions occurs more frequently, however its clinical value is questionable as they are often are asymptomatic [3, 16, 33–36]. Fixation might be considered in a selective group where there are clinical signs of persistent DRUJ instability (ballottement test) after fixation of the radius. Several studies support that a fracture at the ulnar styloid base, including the fovea and TFCC, or with significant displacement (>2 mm) of the fragment increases risk of DRUJ instability [4, 5, 7, 8, 13, 37]. Regrettably, studies included in the present meta-analysis provided limited to no details on clinical instability of the DRUJ after fixation of the radius. Therefore, subgroup analyses could not be performed.

Hardware irritation was the most common complication and frequently resulted in implant removal. It should, however, be acknowledged that in all included studies tension band wiring (TBW) was used as fixation method. Previous studies have shown that this technique leads to more irritation and removal than less prominent implants, such as anchor sutures [38]. Furthermore, headless compression screws are increasingly being used in daily practice for USP fractures. These modern techniques might have a positive effect on hardware complications and mitigate arguments against surgery [39]. Then again, TBW is considered biomechanically superior as it restores both translational and rotational stability [40]. How this balance between biomechanical stability and implant prominence translates into clinical outcomes, remains undefined.

It should be acknowledged that routine post-operative management after surgical fixation of distal radius and USP fractures comprises of functional treatment without the need for plaster casting. However, in the included studies of the present meta-analysis the operatively treated groups received post-operative plaster casting, while generally, early functional mobilisation results in better functional outcomes [41]. Plaster casting after surgery may have negatively affected outcomes in the operative groups and potentially confounded results in favour of the non-operative group. On the other hand it should be noted that in case of persistent DRUJ instability after fixation of the USP and DRF fractures, immobilisation of rotational movement might contribute to DRUJ stability.

In summary, routinely fixating UPS base fractures is not advisable. Surgery should be considered in selective cases such as persistent DRUJ instability after fixation of the radius. When surgery is indicated, low profile implants such as headless compression screws may reduce risk of implant irritation and subsequent removal.

## Limitations

Firstly, one of the studies included base, waist and tip fractures of the ulnar styloid process. However, this did not affect results as there was no difference in the pooled estimate of the primary outcome when this study was removed. Secondly, fracture complexity of the radius also influences functional outcome. It remains unclear how much fracture morphology of the radius confounded the relation between management of the USP base fracture and the measured outcomes in the present meta-analysis. Thirdly, the (quick)DASH questionnaire is not specifically designed for hand and wrist injury but for all upper extremity injury. This may have influenced the reliability of the results for wrist function, compared to when a hand and wrist questionnaire such as the Patient Reported Wrist Evaluation (PRWE) would have been used. Fourthly, the existing amount of evidence is sparse (*n* = 5) and heterogeneity was frequently present amongst included studies. Furthermore, the overall methodological quality of the current existing evidence was moderate, even though two randomized controlled trials were included. Finally, the results are only applicable for long term outcomes with a minimum of six or more months and for general base fractures of the ulnar styloid process, hence results cannot be used to estimate short term effect of treatment modalities or make distinction between subgroups (e.g. DRUJ instability).

## Conclusion

Non-operative treatment results in better wrist function at 6 months after injury, however this difference becomes insignificant after 12 months. Operative management results in more complications, most frequently caused by hardware irritation and subsequent removal. Non-unions occur more frequently in the non-operative group. The clinical relevance of non-unions is, however, questionable as most are asymptomatic. No other significant differences were found regarding outcomes of interest.

Considering these results, routinely fixating USP base fractures as standard of care is not indicated. Rather, surgery should be reserved for selective cases, such as persistent DRUJ instability (positive ballottement test) following radius fixation. Modern fixation methods for primary surgery using less prominent implants might mitigate arguments against surgery. Future research should focus on circumstances warranting surgical intervention and exploration of modern fixation techniques and subsequent complication rates.

## Supplementary Information

Below is the link to the electronic supplementary material.

Supplementary file1 (DOCX 170 KB)

## Data Availability

Data is provided within the manuscript or supplementary information files. Original data files will be preserved by the corresponding author. If necessary data can be provided to third parties. Suppelement data will be available via an online link.
