# Supplementary material for: Operative versus non-operative treatment of ulnar styloid process base fractures: a systematic review and meta-analysis
Source: Eur J Trauma Emerg Surg. 2024 Sep 13;50(6):2843–54. doi: 10.1007/s00068-024-02660-2 (PMC11666621; doi:10.1007/s00068-024-02660-2)
Supplement: Supplementary file 1 — Supplementary file1 (DOCX 170 KB) [file 68_2024_2660_MOESM1_ESM.docx]

**Supplementary material**

| **Table S1**. A detailed overview of the search syntax | | | |
| --- | --- | --- | --- |
| Database | **#** | **Syntax** | **Results** |
| PUBMED/MEDLINE (n = 575) | 1 | ((("ulnar styloid frac*"[Title/Abstract]) OR (“fracture of ulnar styloid”[Title/Abstract]) OR (“distal ulna* frac*”[Title/Abstract]) OR (“metaphyseal ulna*”[Title/Abstract]) OR (“Ulnar styloid base frac*”[Title/Abstract]) OR (“ulnar styloid”[Title/Abstract]) OR (“ulnar base”[Title/Abstract]) OR (“ulnar styloid base” [Title/Abstract]) OR (“ulnar styloid process”[Title/Abstract]) OR (“distal ulna” [Title/Abstract]))) | 1227 |
|  | 2 | (((radius fracture[MeSH Terms]) OR (“radius”[MeSH Terms]) OR ("distal radius fracture*"[Title/Abstract]) OR (“radius fracture*”[Title/Abstract]) OR (“distal radius”[Title/Abstract]))) | 23292 |
|  | 3 | 1 AND 2 | 575 |
| Last update 7-11-2023 | 1 | Original search repeated | 593 |
| EMBASE (n = 185) | 1 | ('ulnar styloid process':ti,ab,kw OR 'ulnar styloid fracture':ti,ab,kw OR 'ulnar styloid':ti,ab,kw) | 559 |
|  | 2 | ('distal radius fracture':ti,ab,kw OR 'radius fracture':ti,ab,kw OR 'distal radius':ti,ab,kw) | 11356 |
|  | 3 | 1 AND 2 | 185 |
| Last update 7-11-2023 | 1 | Original search repeated | 198 |
| CENTRAL (n = 33) | 1 | (‘Ulnar styloid fracture’) | 34 |
|  | 2 | (‘distal radius’) | 14 |
|  | 3 | 1 AND 2 | 33 |
| Last update 7-11-2023 | 1 | Original search repeated | 36 |

| **Table S2.** NEXT-tool critical appraisal of articles comparing operative to nonoperative treatment of ulnar styloid process fractures. | | | | | | |
| --- | --- | --- | --- | --- | --- | --- |
|  | | **RCTs** | | **Observational studies** | | |
| ^a^Items | **^b^Studies** | **Moradi et al. 2021** | **Afifi et al. 2022** | **Zenke et al. 2012** | **Sawada et al. 2016** | **Velmurugesan et al. 2023** |
| PICO | |  | |  | | |
| Item 1 - Population | | 2 | 2 | 2 | 2 | 2 |
| Item 2 - Intervention | | 2 | 2 | 1 | 1 | 2 |
| Item 3 - Comparator | | 2 | 2 | 1 | 1 | 2 |
| Item 4 - Outcome | | 2 | 1 | 2 | 1 | 1 |
| Methodology | |  | |  | | |
| Item 5 - Confounding | | 2 | 2 | 0 | 0 | 0 |
| Item 6 - Missing data and selection bias | | 0 | 2 | 2 | 2 | 1 |
| Item 7 - Intervention status | | 2 | 2 | 2 | 2 | 2 |
| Item 8 - Outcome assessment | | 1 | 1 | 1 | 1 | 1 |
| Item 9 - Pre-specification analysis | | 0 | 2 | 0 | 0 | 0 |
| Total score | | 13 | 16 | 11 | 10 | 11 |
| ^a^Applicability or quality per item is scored as 0 (poor), 1 (moderate) or 2 (good). | | | | | | |
| ^b^Scores range from 0-18 for RCT's and observational studies | | | | | | |

*Figure S1*- Overview of items of NEXT tool and scores per item of included articles
